# Evaluation of Internet-Based Clinical Decision Support Systems

**DOI:** 10.2196/jmir.1.2.e6

**Published:** 1999-11-19

**Authors:** Karl W Thomas, Charles S Dayton, Michael W Peterson

**Affiliations:** ^1^Division of PulmonaryCritical Care and Occupational MedicineUniversity of IowaIowa City IowaUSA; ^2^Department of Medicine and Department of Pharmaceutical CareUniversity of IowaIowa City IowaUSA; ^3^Department of Internal Medicine Internet Education ProjectUniversity of IowaIowa City IowaUSA

**Keywords:** Asthma, Tuberculosis, Decision Support System, Clinical Guidelines

## Abstract

**Background:**

Scientifically based clinical guidelines have become increasingly used to educate physicians and improve quality of care. While individual guidelines are potentially useful, repeated studies have shown that guidelines are ineffective in changing physician behavior. The Internet has evolved as a potentially useful tool for guideline education, dissemination, and implementation because of its open standards and its ability to provide concise, relevant clinical information at the location and time of need.

**Objective:**

Our objective was to develop and test decision support systems (DSS) based on clinical guidelines which could be delivered over the Internet for two disease models: asthma and tuberculosis (TB) preventive therapy.

**Methods:**

Using open standards of HTML and CGI, we developed an acute asthma severity assessment DSS and a preventative tuberculosis treatment DSS based on content from national guidelines that are recognized as standards of care. Both DSS's are published on the Internet and operate through a decision algorithm developed from the parent guidelines with clinical information provided by the user at the point of clinical care. We tested the effectiveness of each DSS in influencing physician decisions using clinical scenario testing.

**Results:**

We first validated the asthma algorithm by comparing asthma experts' decisions with the decisions reached by nonpulmonary nurses using the computerized DSS. Using the DSS, nurses scored the same as experts (89% vs. 88%; p = NS). Using the same scenario test instrument, we next compared internal medicine residents using the DSS with residents using a printed version of the National Asthma Education Program-2 guidelines. Residents using the computerized DSS scored significantly better than residents using the paper-based guidelines (92% vs. 84%; p <0.002). We similarly compared residents using the computerized TB DSS to residents using a printed reference card; the residents using the computerized DSS scored significantly better (95.8% vs. 56.6% correct; p<0.001).

**Conclusions:**

Previous work has shown that guidelines disseminated through traditional educational interventions have minimal impact on physician behavior. Although computerized DSS have been effective in altering physician behavior, many of these systems are not widely available. We have developed two clinical DSS's based on national guidelines and published them on the Internet. Both systems improved physician compliance with national guidelines when tested in clinical scenarios. By providing information that is coupled to relevant activity, we expect that these widely available DSS's will serve as effective educational tools to positively impact physician behavior.

## Introduction

In the last 30 years, we have seen an explosion in basic and clinical research on disease pathophysiology and treatment. Coupled with increased demands on healthcare delivery systems, this rapid growth in scientific knowledge has made the practice of medicine increasingly complex. Local, national, and international organizations have responded to this growing complexity by developing clinical practice guidelines to simplify and improve healthcare quality and delivery. Despite the widespread publication of clinical standards and practice guidelines, however, physicians have had difficulty understanding and applying these guidelines in the clinical care setting. As a result, their practice patterns often do not reflect these consensus-derived, evidence-based recommendations [1-3]. It is clear that in addition to the development and content of clinical practice guidelines, dissemination and implementation strategies are critical to the impact the guidelines will have on physician behavior [4]. Furthermore, the local environment, health care system variables, and patient-specific variables also influence physician acceptance of clinical guidelines [2-5].

Both paper- and computer-based decision support systems (DSS) have evolved to educate physicians about practice standards and to improve guideline impact on a case-specific basis. Computerized DSS's can enhance physicians' clinical performance and guideline compliance in a wide variety of settings [6,7]. When used at the point of clinical care, automated, computer-based DSS's improve physician compliance with specific treatment guidelines [8,9]. In contrast to static, paper-based DSS's, a well-designed, computerized DSS can provide patient-specific information to the user at the time and location of need, at the content level appropriate for the user, and at a pace individualized to the user. Given that adult education occurs most effectively when coupled to relevant activity [10], computerized DSS's incorporated into the workflow of clinical care have the potential to function as important medical education tools.

Many previously described computerized DSS's are proprietary, run on local networks, and are therefore not available to most physicians. While physicians have not yet widely embraced the Internet as a professional information source, they are nevertheless beginning to use it for clinical information and education [11,12]. Because of its open standards, the Internet can be used to deliver information easily to computer networks anywhere in the world. The Internet therefore has the immense potential to serve as an educational resource and dissemination tool for clinical guidelines and DSS's. This potential is contingent on developing tools that are openly and freely available, can be adapted to local conditions, and can be incorporated into the physician workflow.

In this study, we report our initial experiences in the development of two DSS's delivered over the Internet. We developed a DSS for the National Asthma Education Program-2 (NAEP-2) [13] Asthma Treatment Guidelines, and another for the American Thoracic Society/Centers for Disease Control (ATS/CDC) Tuberculosis Preventive Guidelines [14], using Hypertext Markup Language (HTML) and Common Gateway Interface (CGI) open standards. We then tested the effectiveness of the Internet-delivered guidelines compared to paper-based resources using clinical scenario testing.

## Methods

### General Design Considerations

During the development of both DSS's, we incorporated several design characteristics that have been identified as important for widespread acceptance and utilization [15]. These characteristics included telegraphic representations of the guidelines, navigation components commonly used on the World Wide Web (WWW), clear indexing, and forgiving interfaces. Furthermore, each DSS was designed to operate with input from clinicians entered with mouse clicks; neither requires free text or detailed data entry. Both DSS's were designed to function as supplements and additions to existing national guidelines. We provided these guidelines in their entirety within each system through HTML links.

### Development of the Acute Asthma Severity Evaluation and Treatment Decision Support System

**Figure 1:**
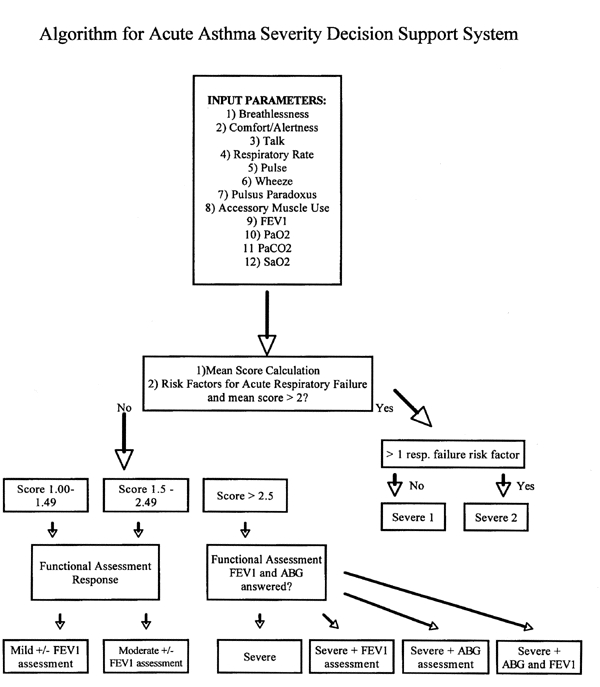
Algorithm For Acute Asthma Severity Decision Support System

The NAEP-2 clinical asthma guidelines form the knowledge base upon which we structured the asthma DSS. We created the acute asthma DSS to complement and expand the existing guidelines by providing specific guideline information tailored to a unique clinical situation. The asthma DSS provides the clinician with information on disease severity assessment, recommendations for objective functional patient testing, and recommendations for case-based treatment. Furthermore, the DSS serves as a portal for hypertext navigation through the entire NAEP-2 guidelines. We organized the operation of the DSS on a simple decision tree model for acute asthma exacerbations (Figure 1).

The DSS first estimates disease severity by calculating an arithmetic mean of up to 12 scores of clinical parameters provided by the user. The input parameters cover asthma symptoms, signs, and objective findings as assessed and interpreted by the clinician. The algorithm also identifies risk factors for acute respiratory failure and determines whether objective assessments, e.g. arterial blood gases or pulmonary function tests, are indicated based on disease severity. Using CGI, we scripted this algorithm for use on the Internet and published it on the WWW at the Virtual Hospital(tm) at the following URL: 

http:/www.vh.org/Providers/ClinGuide/AsthmaIM/Default.html

### Development of the ATS/CDC Preventive Tuberculosis Guidelines Decision Support System

Using a similar approach based on hypertext links, we developed a second DSS for treating positive tuberculosis skin reactions. We organized the ATS/CDC guidelines for preventive tuberculosis care into a series of hierarchical choices based first on the size of the reaction to purified protein derivative (PPD) and then on specific patient characteristics. Using these two sets of input parameters to drive the decision process, we created a series of individual information pages using HTML to be delivered on the WWW. We published this DSS along with the hypertext-linked pages containing background information and the ATS/CDC guidelines themselves on the Virtual Hospital(tm) at the following URL: 

http:/www.vh.org/Providers/TeachingFiles/PulmonaryCoreCurric/TBCase/AssessmentTool/AssessmentPage1.html

### Development and Scoring of Clinical Case Scenarios

We tested both the asthma and tuberculosis DSS's with case scenarios. Using the NAEP-2 guidelines as the primary resource, we developed and refined six acute asthma scenarios to reflect mild, moderate, and severe exacerbations. Each case scenario also included information on the response of the hypothetical patient to the proposed treatments. Five questions reflecting the main points of the NAEP-2 guidelines followed each scenario: 1) initial assessment choices, (mild, moderate, severe, or severe with life-threatening features); 2) diagnostic testing requirements (peak flow, arterial blood gas analysis); 3) initial treatment choices (b-agonists, corticosteroids, oxygen, cholinergic antagonists, methylxanthines, and antibiotics); 4) treatment response assessment (poor, incomplete, or good response); and 5) patient disposition (discharge to outpatient management, inpatient general ward, or inpatient ICU). The same questions followed each scenario. We scored the responses to the clinical scenarios using the NAEP-2 guidelines as the standard for the most appropriate response. Responses generated the maximum possible score if they reflected the guidelines; partial credit was given for answers near the correct answers (e.g. moderate rather than severe assessment). The maximum possible score was the same for each question in each case scenario.

To test our second DSS, we developed eight wide-ranging patient scenarios of tuberculosis infection. Four local tuberculosis experts validated the DSS by assuring that the recommendation provided by the DSS was the same as the recommendation provided by the ATS/CDC guidelines. All four experts agreed that the DSS recommendation for the each of the eight scenarios accurately reflected the ATS/CDC guidelines. We scored the responses of the test groups on the basis of agreement with the ATS/CDC guidelines.

### Testing the Decision Support Systems

Four groups of healthcare practitioners completed the acute asthma case scenarios. The first group included board-certified pulmonologists (N=10), who were asked to complete the questions based on their practice patterns. This group was our positive control group to verify the system. The second group consisted of clinical oncology nurses who work with respiratory patients but have no special expertise in asthma (N=5). They answered the same scenario questions with the assistance of the acute asthma DSS. Medical residents (postgraduate training year 1 to 3) at the University of Iowa Hospital comprised the third and fourth test groups in the asthma DSS evaluation. We randomly assigned an unselected group of residents attending a teaching conference to complete the scenarios using either the DSS (N=11) or a printed copy of the Practical Guide for the Diagnosis and Management of Asthma [16] provided with the scenarios (N=16).

We developed this DSS using open standards to make it easier for physicians to use the program. We reasoned that physicians would be more likely to use the tools if they did not require additional training. For that reason, we did not provide instruction in the use of the DSS prior to the scenario testing. In this way, we could determine whether the system was intuitive and easy to use.

We similarly tested the tuberculosis DSS using a group of unselected general Internal Medicine Residents (postgraduate training year 1 to 3) at the University of Iowa Hospital. The evaluation occurred during a regularly scheduled teaching conference with no prior selection of participating residents. We randomly assigned residents to answer the same eight clinical scenario questions using the computerized DSS (N=12) or using written resources (N=17). The written resource consisted of a guideline card developed at our institution, which was provided to all participants. For the same reason as above, we did not provide any specific training in the use of the tuberculosis DSS prior to the scenario testing.

### Statistical Analysis

After establishing normality, the mean total scores and individual components of the scores for the tuberculosis and acute asthma clinical scenarios were analyzed with two-sample t tests. Cronbach's coefficient alpha was calculated for the acute asthma clinical scenarios as a measure of internal consistency of the testing instrument. We used SPSS v. 9.0 software (SPSS Inc., Chicago IL, 1998) for all data analysis.

## Results

### Acute Asthma DSS

We first evaluated the validity of the asthma clinical scenarios by analyzing the consistency of the responses. Cronbach's standardized coefficient alpha calculated for all the asthma scenarios was 0.76, indicating good internal consistency of the scenario testing instrument. When we recalculated the intraclass correlation coefficient after deleting each item of the test instrument sequentially, we found good reliability with an alpha range 0.67 to 0.71.

**Figure 2:**
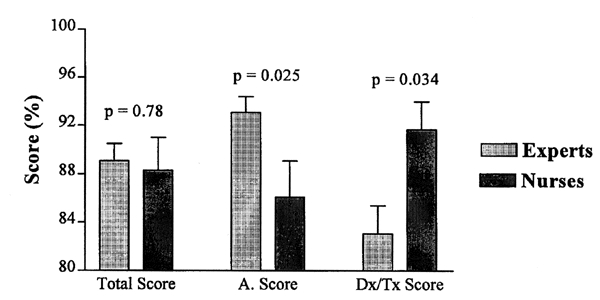


As noted in the previous section, four groups of participants completed the acute asthma scenario test instrument as described in materials and methods. The expert panel (N=10), instructed to answer the questions based on their practice standards, had a mean score of 89.1% (95% CI 86.0-92.1%) in agreement with the guidelines. The group of clinical nurses (N=5) with no respiratory specialty training who used the acute asthma DSS reached a mean score of 88.3% (CI 80.9-95.8%). The nurses' performance was not statistically different from the experts at a p = 0.78 (Figure 2). These data demonstrate that a group of health professionals without specific training in asthma can reach the same conclusions when using the Internet DSS as experts.

**Figure 3:**
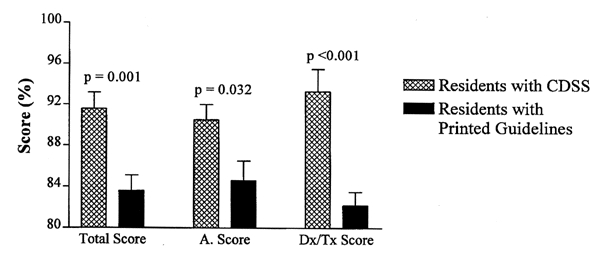


We next examined the effect of the DSS on the decision-making process among similarly trained physicians. An unselected group of Internal Medicine residents was randomly divided and assigned to use either the DSS (N=11) or a printed copy of the Practical Guide for the Diagnosis and Management of Asthma [16]. The residents using the DSS performed much better than the residents using the printed guidelines (mean score 91.6%, CI 88.0-95.3% compared to 83.6%, CI 80.5-86.7%; p = 0.001) (Figure 3). There was no statistical difference between the mean scores of the expert panel and the residents using the DSS (p = 0.26). However, the residents using only the printed materials performed statistically worse than the experts (p = 0.017). Both the resident group using the computer DSS and the resident group using the printed resources completed all patient scenarios in less than 30 minutes (% minutes per case).

When we analyzed the specific components of the asthma scenario questions, we found that the expert panel performed better than the nurses on initial and repeat asthma severity assessment (93% vs. 86%, p = 0.025). This difference was offset by the expert group's tendency to order more diagnostic tests, to hospitalize more frequently, and to treat some cases more aggressively than the nurses using the treatment guidelines delivered through the DSS (83% correct vs. 92%, p = 0.034) (Figure 2). In a similar analysis of the resident groups, the cohort using the DSS performed better in all areas tested including severity assessment and diagnostic/therapeutic responses than did the residents using printed guidelines (p = 0.03 and p < 0.005 respectively) (Figure 3).

### ATS/CDC Preventive Tuberculosis DSS

We compared the effect of printed guideline reminders to the computerized DSS for preventative tuberculosis treatment in a separate session of scenario testing. Two randomly assigned groups of unselected Internal Medicine residents ranging from postgraduate years 1 to 3 participated in this assessment of responses to eight clinical scenarios. The computer-based group (N=12) demonstrated better compliance with the ATS/CDC guidelines on each scenario than did the paper-based resource group (N=17). Overall, the computer group reached the appropriate ATS/CDC recommendation in 92/96 scenarios or 95.8%, (8 scenarios x 12 subjects in the group). The medical resident group using the paper resource, however, reached the same conclusion as the ATS/CDC guidelines in only 77/136 or 56.6%, (8 scenarios x 17 subjects in the group). This difference was statistically significant at p < 0.001. The computer group required less than 2 minutes per case to reach their conclusions.

## Discussion

The profession of medicine has a long history of continuing education in an attempt to assure that all physicians are practicing the highest quality medicine [17]. In the last few decades, this pursuit has contributed to the development of evidence-based medicine and to a proliferation of practice guidelines [18]. Unfortunately, many studies have demonstrated that traditional continuing education, including lectures and the publication of practice guidelines, does not significantly alter physician behavior. This ineffectiveness may result from the fact that these modalities fail to link the educational activity to the time and setting of the intended activity. DSS's are one method to overcome this shortfall. DSS's can act to remind physicians of certain behaviors at the most appropriate time and location. They are, therefore, more likely to serve as effective educational tools. Both paper-based and computer-based DSS's can change physician behavior and improve quality of care. In contrast to paper-based clinical support systems, computerized DSS's can easily accommodate broad content and knowledge bases that can be accessed using specific clinical information quickly and efficiently.

Many issues including physician reluctance, proprietary interests, technical limitations, and local practice environments limit the widespread application of many previously reported computer-based DSS's [19,20]. The growth and availability of the Internet provides a new model for sharing medical knowledge and DSS's across existing computer networks. The main limitations of the Internet include technical aspects imposed by the open standards and the challenge of controlling information quality [21,22]. These limitations, however, do not necessarily impose disadvantages. The open standards available on the Internet allowed us to simplify our user interface within existing standards and thus ensure widespread availability and ease of navigation. To target the largest possible audience, we used nationally developed and widely applied clinical guidelines. Therefore, we were able to restrict the focus of our data synthesis and delivery to peer-reviewed information of the highest quality.

We developed DSS's and published them on the Internet for two medical problems, asthma management and tuberculosis preventive therapy. We chose these problems to test for several reasons. First, high quality and well-accepted standards are available for both problems. Second, we identified both these subject areas as important for clinical improvement. We have previously found that physicians have difficulty applying the National Asthma Education Program's staging system and treatment recommendations to patients [1]. Our results are not unique; other investigators have identified significant deviations in asthma care from the guidelines in many different settings [23,24]. Divergent clinical practice patterns with respect to published guidelines for tuberculosis treatment have been similarly described [25]. Finally, both of these problems are exceedingly common around the world and their increasing incidence presents a significant management challenge for clinicians.

Before widespread clinical application of our DSS's, we sought to test the effectiveness and accuracy of both computerized systems in relevant but controlled artificial clinical settings. We evaluated both systems using scenario testing, a technique which has been established and used successfully to test clinical decision-making [26]. When compared to the performance of experts, both DSS's produced accurate and reliable responses reflective of evidence-based standards of care. The acute asthma DSS assisted both medical residents and clinical nurses to perform at levels not statistically different from a group of experienced pulmonologists. Similarly, the Internet-based tuberculosis DSS delivered the same responses as a panel of experts, and improved medical resident compliance with ATS/CDC recommendations for treating PPD reactions.

One interesting finding with the asthma DSS was the fact that inexperienced nurses did not score as well as experienced pulmonologists in certain subcategories but performed better in others. We did not find similar differences in the two resident groups. This suggests that the computer DSS works most consistently and accurately when used by more experienced clinicians. The design of each DSS around national guidelines that provide generic information which must be interpreted for unique patients requires that the physician provide his or her own judgment before clinical action is taken. Thus, the DSS supplements but does not replace human clinical judgment. Furthermore, by providing relevant information at the point of need, these DSS's can function to educate and encourage sustained improvements in clinical judgment.

Finally, we have developed the acute asthma and preventative tuberculosis DSS using open standards for information display, HTML and CGI, thus enabling them to be published on the WWW. These systems function independently of local patient data systems by using an interactive format, which requires the input of clinical parameters by the user. While this design characteristic does create some barriers to automatic DSS function through an interface with local computerized patient records, it also avoids numerous technical database management and non-uniform data exchange issues. By requiring only that the user have Internet access, these nonproprietary decision support tools are widely available with existing technology. Given the fact that increasing numbers of physicians are using computers and the Internet as a source of medical information [11], we expect that Internet accessibility will enhance DSS use and integration into physician workflow.

In summary, we have published two computerized decision support tools on the WWW and evaluated them using clinical scenario testing. We have shown that both guideline-based DSS's delivered over the Internet enabled the users to more accurately choose assessment or treatment plans in concordance with established guidelines. While patient outcomes with these systems have not yet been evaluated, we expect that the widespread availability and adaptability to local conditions of these systems will further guideline dissemination and implementation.
